# Interaction between ROS dependent DNA damage, mitochondria and p38 MAPK underlies senescence of human adult stem cells

**DOI:** 10.18632/aging.100673

**Published:** 2014-06-12

**Authors:** Aleksandra Borodkina, Alla Shatrova, Polina Abushik, Nikolay Nikolsky, Elena Burova

**Affiliations:** ^1^ Department of Intracellular Signaling and Transport, Institute of Cytology, Russian Academy of Sciences, St. Petersburg, Russia; ^2^ Sechenov Institute of Evolutionary Physiology and Biochemistry, Russian Academy of Sciences, St. Petersburg, Russia; ^3^ Department of Medical Physics, St.Petersburg State Polytechnical University, St Petersburg, Russia

**Keywords:** mesenchymal stem cells, ROS, DDR, senescence

## Abstract

Human endometrium-derived mesenchymal stem cells (hMESCs) enter the premature senescence under sublethal oxidative stress, however underlying mechanism remains unknown. Here, we showed that exogenous H_2_O_2_ induces a rapid phosphorylation and co-localization of ATM, H2A.X, 53BP1 leading to DNA damage response (DDR) activation. DDR was accompanied with nuclear translocation of p-p53 followed by up-regulation of p21Waf1 and the permanent hypophosphorylation of pRb. Additionally, the increased p38MAPK/MAPKAPK-2 activation persisted in H_2_O_2_-treated cells. We suggest that both p53/p21/pRb and p38MAPK/MAPKAPK-2 pathways are responsible for establishing an irreversible cell cycle arrest that is typical of senescence. The process of further stabilization of senescence required prolonged DDR signaling activation that was provided by the permanent ROS production which in turn was regulated by both p38MAPK and the increased functional mitochondria. To reverse senescence, the pharmacological inhibition of p38MAPK was performed. Cell treatment with SB203580 was sufficient to recover partially senescence phenotype, to block the ROS elevation, to decrease the mitochondrial function, and finally to rescue proliferation. Thus, suppression of the p38MAPK pathway resulted in a partial prevention of H_2_O_2_-induced senescence of hMESCs. The current study is the first to reveal the molecular mechanism of the premature senescence of hMESCs in response to oxidative stress.

## INTRODUCTION

Cellular senescence defined as an irreversible proliferation arrest promotes age-related decline in mammalian tissue homeostasis [1]. At present, investigation of this phenomenon becomes more and more widespread due to the following reasons: firstly, senescence is thought to contribute to a multiple age-related pathologies [2, 3], and secondly, senescence acts as a tumor suppressor mechanism that is able to block proliferation of incipient cancer cells [1, 4]. Similar to other types of normally proliferating cells that are characterized by a finite lifespan (Hayflick limit) human mesenchymal stem cells undergo the replicative senescence after a fixed number of cell divisions [5].

Moreover, the recent findings have revealed that human mesenchymal stem cells may respond to a variety of subcytotoxic stresses (UV-, γ-radiation, H_2_O_2_, histone deacetylase inhibitors, etc.) by induction of premature senescence [6, 7, 8].

Oxidative stress has been shown to play an important role for the development of aging and age-related diseases [9]. According to the free-radical theory of aging, reactive oxygen species (ROS), including the oxygen singlet, the superoxide anion (O2^.-^), the hydroxyl radical (OH^.^) and hydrogen peroxide (H_**2**_O_**2**_) might be the candidates, which are responsible for cellular senescence. Being membrane permeable and long-lived molecule, H_2_O_2_ can directly affect the cellular DNA, inducing both single- and double-strand breaks (SSBs and DSBs, respectively) [10]. DNA damage, in turn, triggers a specific DNA damage response (DDR), which involves the following events – (1) activation of any sensor kinases (ATM, ATR, DNA-PK), (2) phosphorylation of adaptor protein 53BP1, and (3) formation of the discrete foci, containing phosphorylated histone H2A.X and p53BP1 [11]. Finally, DDR activation leads to cell cycle arrest via activation of p53/p21 [12] and/or p16/pRb pathways [13, 14]. Today it is generally accepted that both the replicative and stress-induced senescence are the outcome of DDR [15].

Conversion from proliferative arrest to irreversible senescence, a process named geroconversion, is driven in part by growth-promoting pathways in particular mammalian target of rapamycin (mTOR) which is mostly responsible for loss of RP (replicative/regene-rative potential) and hypertrophy [16, 17]. Inhibitors of mTOR such as rapamycin [18] and hypoxia [19] can suppress geroconversion, maintaining quiescence instead. Previously, the mTOR was the only pathway known to be involved in acquiring classic markers of a senescent phenotype, including cyclin D1 accumulation. Recent studies have been revealed an additional MEK/ERK pathway that is required for the acquisition of at least one hallmark of senescence: hyper-accumulation of cyclin D1 [20]. Furthermore, it was shown that p70S6K, a crucial substrate of mTOR, and MEK play different roles in geroconversion [21].

According to recent publications, a so-called feedback loop between the permanent DDR activation and the increased ROS production is necessary for development of senescence [22]. The main effectors of DDR, p53 and p21, were shown to be involved in regulation of ROS generation, leading to enhanced intracellular ROS during the establishing of senescence [23, 24]. In the senescent cells, elevated ROS can cause a direct DNA damage and the persistent DDR activation, thereby forming a feedback loop.

It is well-established that the senescent cells are characterized by increased ROS levels. Taken into consideration that the overwhelming majority of intracellular ROS are of mitochondrial origin, it is reasonable to posit that the elevated ROS production might be caused by alteration in mitochondrial function during senescence. Mitochondria are the intracellular organelles responsible for ATP synthesis through the coupling of oxidative phosphorylation to respiration in mammalian cells. Currently, there are different points of view regarding age-related changes in mitochondrial physiology. Several authors considered cellular senescence to be accompanied by mitochondrial dysfunction defined by the decline of mitochondrial membrane potential (MMP). MMP decline leads to respiratory-chain defects and thus to enhanced ROS production [22, 25]. On the contrary, the others hypothesized the absence of defects either in electron transport system or in oxidative phosphorylation during senescence [26, 27]. In this case, ROS levels are elevated due to (1) the growing number of functional mitochondria and, (2) the age-related alterations in mitochondrial coupling that correlates with the increase in mitochondrial membrane potential. Importantly, both hypotheses manifested the involvement of mitochondria in the aging process via a relevant contribution to intracellular ROS generation.

p38, a member of the family of mitogen-activated protein kinases (MAPKs) is activated by cellular stresses including ROS, UV- and γ-radiation, and proinflammatory cytokines [28]. In response to stress factors, p38 MAPK (hereafter p38) can rapidly phosphorylate and activate MAP kinase-activated protein kinases (MAPKAPKs), particularly MAPKAPK-2 (hereafter MK-2) that is a direct target of p38 [29]. The p38 effector kinase lies downstream of the MKK3/6 activator kinases, whose activity can be regulated by stress-sensitive apoptosis signal-regulating kinase-1 (ASK1) [28]. p38 plays an important causative role in cellular senescence induced by oxidative stress, radiation, genotoxic agents, Ras overexpression [30]. p38 has been demonstrated to participate in feedback relationships during senescence as, on the one hand, it can function as mediator of ROS signaling and, on the other hand, can directly phosphorylate p53 [22]. The significance of p38 MAPK pathway in stress-induced senescence was investigated predominantly in the cultures of either human fibroblasts [22, 31] or transformed cells [32]. However, there is no information available as to whether the functional p38 is required for premature senescence of human endometrium-derived mesenchymal stem cells (hMESCs).

hMESCs are a relatively new source of adult stem cells intensively studied over the past decade. The fact that hMESCs isolation does not require invasive and traumatic procedures facilitates their use in regenerative medicine. So to date, promising results concerning the experimental and clinical application of these cells for treatment of heart failure, myocardial infarction, diabetes, stroke, Parkinson's disease, multiple sclerosis, Duchenne Muscular Dystrophy and infertility were obtained [33, 34, 35]. Despite the different nature of these disorders, namely oxidative stress is well known to play an essential role in their progression. The goal of the present study was to clarify the underlying mechanisms of both induction and further maintenance of premature senescence in hMESCs subjected to oxidative stress.

## RESULTS

Recently, we have provided the reliable evidence that hMESCs undergo the premature senescence in response to the sublethal concentration of H_2_O_2_ [36]. H_2_O_2_-treated hMESCs were permanently arrested, lost Ki67 proliferative marker, and exhibited senescent phenotype, including cell hypertrophy and increased SA-β-Gal activity, indicating that the cells were driven into stress-induced premature senescence. However, the molecular mechanism of senescence induction in hMESCs under oxidative stress is far from being elucidated.

### DDR activation in response to a rapid accumulation of exogenous H_2_O_2_ in hMESCs

**Figure 1:**
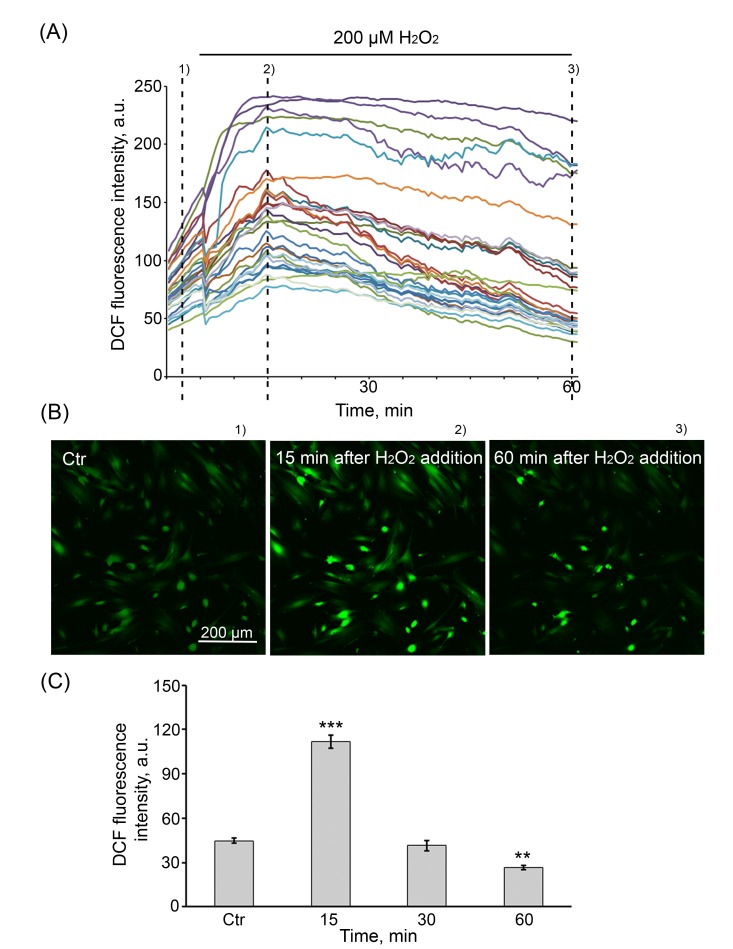
The dynamics of H_2_O_2_ penetration into hMESCs (**A**) Intracellular ROS levels upon application of 200 µM H_2_O_2_. Cells were loaded with DCF, each trace represents the response of one cell. Three (*N* = 3) experiments were performed. (**B**) Fluorescent images taken at different stages (indicated by dashed lines) of the experiment illustrated in (A). Scale bar is 200 µm and valid for all images. (**C**) Intracellular ROS levels measured by FACS after staining with the fluorescent probe DCF. Values are means ± SD of three independent experiments. **p<0.005, ***p<0.001 versus control.

H_2_O_2_ by conventional diffusion may easily pass through the membrane into the intracellular space, causing damage to lipids, proteins, and DNA [37, 38]. To ascertain the dynamics of H_2_O_2_ penetration into the cells in our experimental conditions (200 µM H_2_O_2_, 1 h), in H_2_DCFDA-stained cells, the changes in H_2_O_2_ concentration were monitored for 60 min by either FACS or the confocal microscopy. Of note, the confocal microscopy enabled to estimate the individual changes of fluorescence occurring in each of the cells selected to test (Fig. 1 A, B), whereas FACS analysis evaluated the average fluorescence per cell (Fig. 1 C). Despite the apparent distinctions in fluorescence levels presented in Figures 1A and 1C, resulting from the behavior of individual cells in the heterogenous population, the dynamics of H_2_O_2_ diffusion evaluated by both methods were alike. H_2_O_2_ treatment of cells led to a rapid elevation of intracellular ROS levels, peaking at 15 min and returning progressively to the baseline by 30 min in majority of cells, indicating that exogenous H_2_O_2_ is almost completely utilized by cells during 1 h treatment.

Among intracellular ROS, which are able to induce DNA damage, H_2_O_2_ is known to provoke an appearance of both SSBs and DSBs that can trigger DDR [38]. Generally, DDR is characterized by activation of ataxia-telangiectasia mutated kinase (ATM) and formation of DNA-damage foci, containing γH2A.X and p53BP1 in chromatin, surrounding DSBs. Presently, it is generally accepted that γH2A.X, as well as p53BP1 are recognized as the reliable markers of DSBs [11]. To examine the possibility of DDR activation in H_2_O_2_-treated hMESCs, the functional status of key proteins involved in DDR was estimated.

**Figure 2:**
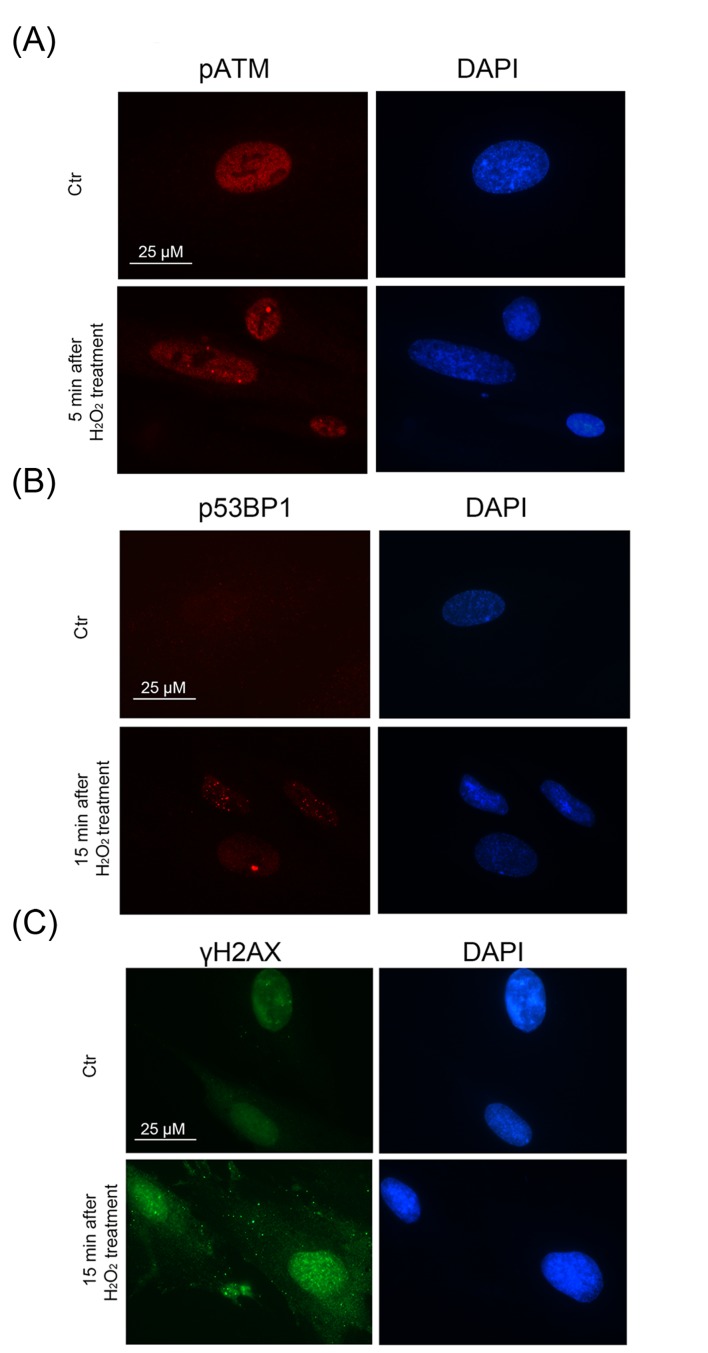
A rapid DDR activation in H_2_O_2_-treated hMESCs Immunofluorescence analysis of ATM (**A**), 53BP1 (**B**) activation and γH2AX foci formation (**C**) performed at indicated time points after H_2_O_2_ addition. DAPI was used as nuclear stain (blue). Representative photomicrographs of the staining are made at original magnification X100. Scale bar is 25 µm.

**Figure 3:**
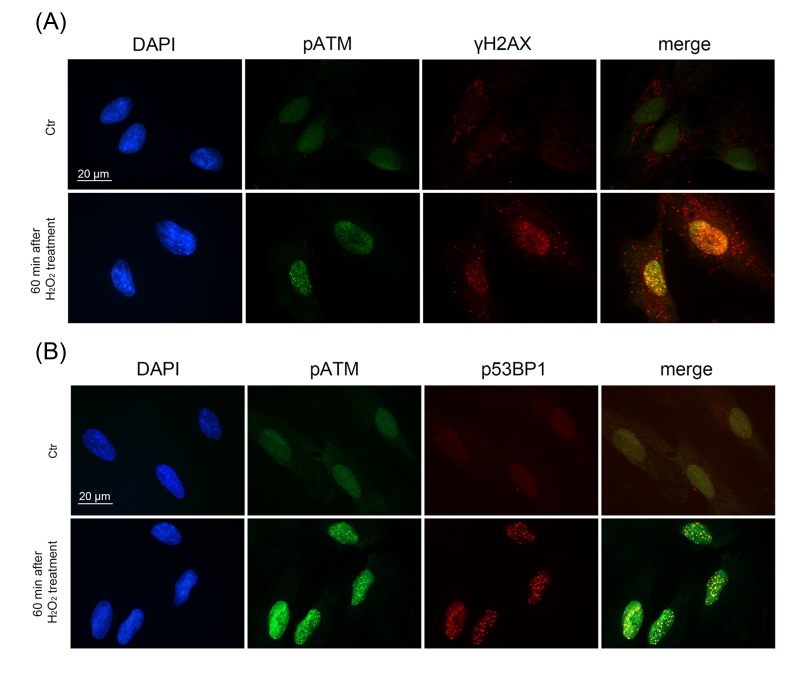
Formation of DNA damage foci containing pATM, γH2AX, p53BP1 in H_2_O_2_-treated hMESCs Immunofluorescent analysis with the use of specific antibodies against pATM, γH2AX, p53BP1 revealed co-localization of pATM with either γH2AX (**A**) or p53BP1 (**B**) in 60 min after beginning of H_2_O_2_ treatment. DAPI was used as nuclear stain (blue). Images are taken at magnification X100. Scale bar is 20 µm and valid for all images.

**Figure 4:**
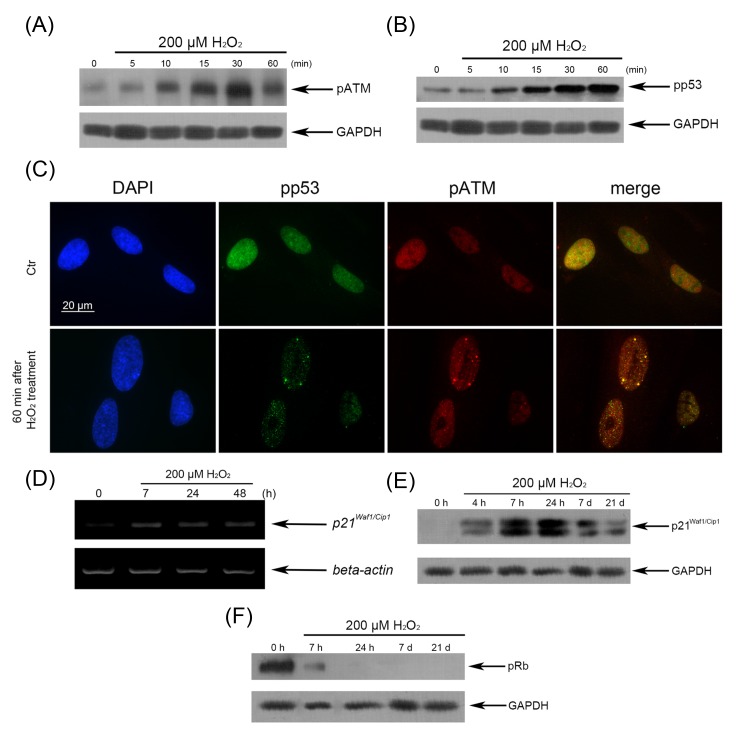
H_2_O_2_-induced activation of the p53/p21/pRb pathway in hMESCs (**A**) H_2_O_2_-induced ATM phosphorylation tested by Western blotting. (**B**) Phosphorylation status of p53 in both untreated and H_2_O_2_-treated cells examined by Western blotting. (**C**) Nuclear accumulation of pp53 and co-localization pp53 with pATM revealed by immunofluorescence. Original magnification, X100. Scale bar, 20 µm. (**D**) The expression levels of *p21* mRNA. (**E**) The expression levels of p21 protein. (**F**) Abrogation of Rb phosphorylation in H_2_O_2_-treated hMESCs examined by Western blotting. GAPDH and β-actin were used as loading controls. Representative results of three independent experiments are shown.

Immunofluorescent analysis with the use of specific antibodies against pATM, γH2A.X, p53BP1 revealed a rapid ATM phosphorylation (within 5 min after beginning of treatment) (Fig. 2 A) and further simultaneous phosphorylation of 53BP1 and H2A.X in 15 min (Fig. 2 B, C). Moreover, we observed co-localization of pATM with either γH2A.X or p53BP1 in 60 min after beginning of treatment (Fig. 3 A, B). The fast H_2_O_2_-induced ATM phosphorylation peaking at 30 min was confirmed by Western blot analysis (Fig. 4 A). Taken together, the results obtained demonstrate that the exogenous H_2_O_2_ was able to cause an immediate DNA damage followed by DDR activation.

### The p53/p21/pRb pathway orchestrates both establishing and maintaining H_2_O_2_-induced senescence of hMESCs

In previous study, we have shown that an irreversible cell cycle arrest is the main marker of H_2_O_2_-induced premature senescence of hMESCs, however the signaling pathways providing the cell cycle arrest require the detail investigation. Senescence program is thought to be developed as result of DDR, leading to functional activation of the p53/p21 pathway, which can establish and maintain the growth arrest [12]. In order to determine whether cell cycle block may be realized via p53/p21 pathway in H_2_O_2_-treated hMESCs, we first investigated the functional status of p53 protein. Western blot analysis with using rabbit polyclonal antibodies against phospho-p53 at Ser15 revealed a rapid (within 10 min) p53 phosphorylation, gradually increasing during 60 min of H_2_O_2_ treatment (Fig. 4 B). At the same time, translocation of phospho-p53 into the nuclei and its partial colocalization with pATM was observed (Fig. 4 C). In H_2_O_2_-treated cells, enhanced p53 phosphorylation was also detected at 7 h post-treatment, however, over next 8 days it was dramatically diminished (Fig. 8 C). Remarkably, the decrease of the functional activity of p53 had no effect on elevated p21 induction during the entire observation period (Fig. 8 D).

It is known that p53 activated acts as a transcription factor, inducing expression of p21 which may mediate the initiation of the cell cycle arrest by inhibiting various cyclin-dependent kinases (CDK) that contribute cell cycle phase progression. Therefore, we next examined mRNA and protein expression levels of p21. H_2_O_2_ promoted a significant elevation in mRNA and protein expression of p21 already at 7 h post-treatment (Fig. 4 D, E). An inducible expression of p21 was up-regulated, at least, during 7 days with following decline to insignificant, but not the control levels, which persisted up to 21 days. The elevated p21 expression was accompanied with the cell cycle arrest at the same time (data not shown). Retinoblastoma protein (pRb) whose activity is regulated by elevated p21 plays a crucial role for establishing the growth arrest. It is known that pRb in active hypophosphorylated state halts cell proliferation by suppressing the activity of E2F transcription factor that regulates cell cycle progression. To examine the functional status of pRb during establishing senescence, we performed monitoring the kinetics of pRb activation in H_2_O_2_-treated hMESCs. As expected, beginning 7 h post H_2_O_2_ treatment, no pRb phosphorylation was observed in the senescent cells, in contrast to the control proliferating cells, which displayed the high levels of pRb phosphorylation (Fig. 4 F). Collectively, our findings demonstrate that the p53/p21/pRb signaling pathway leading to the growth arrest is required to drive the premature senescence and apparently to maintain the long-term senescent state in hMESCs.

### An interplay between enhanced ROS levels and prolonged DDR activation

**Figure 5:**
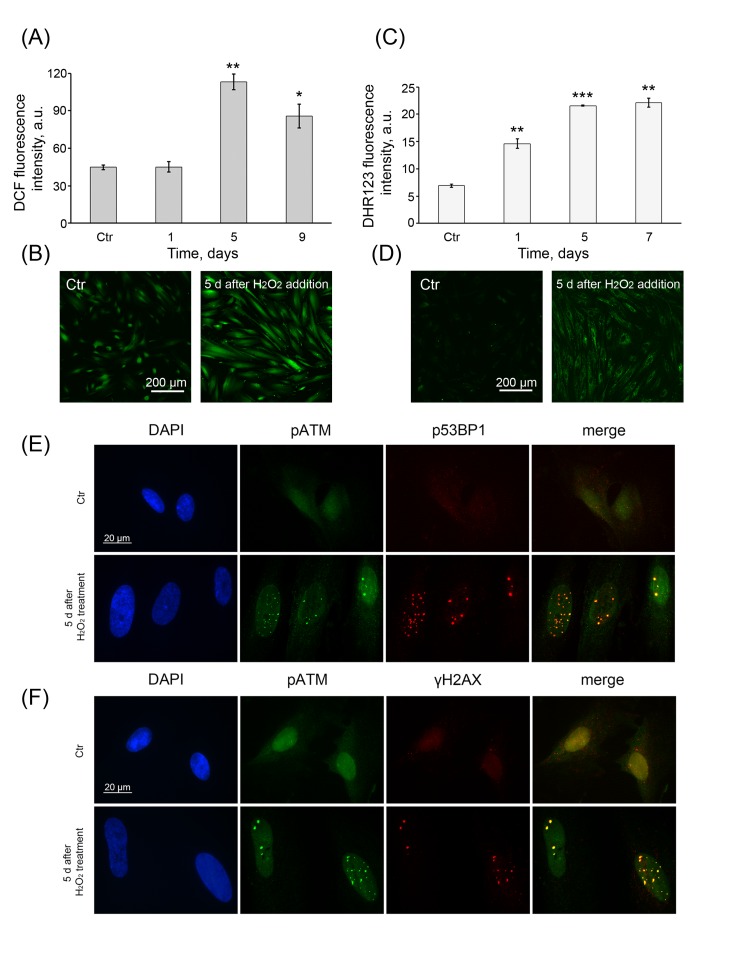
Permanent ROS generation and prolonged DDR activation (**A**) Intracellular ROS levels and (**C**) cellular peroxide levels detected at indicated time points by FACS analysis after staining with either DCF or DHR123, respectively. (M ± SD, *N* = 3, *p<0.05, **p<0.005, ***p<0.001, versus control). Representative DCF (**B**) or DHR123 (**D**) fluorescent images of control (Ctr) and treated hMESCs. Scale bar is 200 µm and valid for all images. (**E**) Co-localization of pATM with either p53BP1 or (**F**) γH2AX in 5 days after H_2_O_2_ treatment. DAPI was used as nuclear stain (blue). Representative photomicrographs of the staining are shown. Images are taken at magnification X100. Scale bar, 20 µm.

As mentioned above, the exogenous H_2_O_2_ induced a strong increase in intracellular ROS levels within 1 h of cell treatment (Fig. 1 A, C) and accordingly triggered a premature senescence of hMESCs. To find out whether the intracellular ROS levels can be modulated during the senescence development, DCF fluorescence intensity was measured in H_2_O_2_-treated cells over the next 9 days. Surprisingly, on day 5 post-treatment, the senescent cells were characterized by strongly increased DCF fluorescence, consistent with higher levels of intracellular ROS that remained elevated further over 9 days (Fig 5A, B). These results were in agreement with the continuous elevated levels of intracellular peroxides measured by DHR123 in the senescent cells (Fig. 5 C, D). These findings clearly demonstrate that the process of H_2_O_2_-induced senescence of hMESCs is accompanied with the permanent generation of the intracellular ROS.

Previous studies have reported that there is the functional link between enhanced ROS production and DDR activation during the development and stabilization of senescence [22]. Therefore, we further characterized the functional status of DDR in the senescent cells by testing ATM, H2A.X and 53BP1 for their phosphorylation and an intracellular localization using the fluorescent microscopy. Remarkably, on 5 days post-treatment all of proteins tested remained in an active state and mostly co-localized in so-called senescence-associated DNA-damage foci (SDFs) (Fig. 5 E, F). It should be noted that, in the senescent cells, enhanced ROS production and DDR activation has been contemporized. Together, these observations allow us to suspect that enhanced intracellular ROS could be responsible for long-term DDR activation.

### An increase in mitochondrial activity in the senescent hMESCs

**Figure 6:**
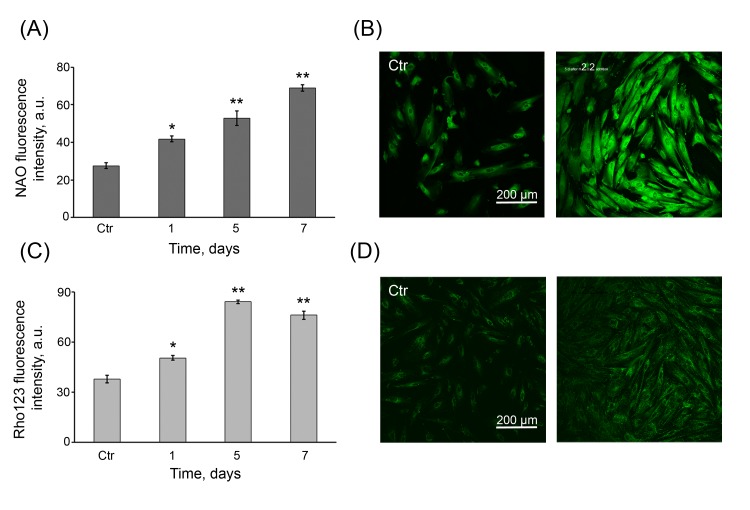
Persistent increase in the number of functional mitochondria in H_2_O_2_-treated hMESCs (**A**) FACS analysis of mitochondrial mass in control (Ctr) and H_2_O_2_-treated hMESCs stained with NAO. Values are means ± SD of three independent experiments (*p<0.05, **p<0.005, versus control). (**B**) Representative NAO confocal fluorescence images of control (Ctr) and treated hMESCs. (**C**) Rho123 fluorescence in hMESCs at the indicated time points after H_2_O_2_ treatment as measured by FACS. (M ± SD, *N* = 3, *p<0.05, **p<0.005, versus control). (**D**) Rho123 fluorescent images of control (Ctr) and H_2_O_2_-treated hMESCs. Micrographs are representative for three experiments. Scale bar is 200 µm and valid for all images.

Although we have shown that permanently enhanced ROS are typical of the senescent state of hMESCs, nevertheless the actual reason of long-term ROS production remained unclear. We hypothesized that this phenomenon might be associated with the significant modulation of the mitochondrial function in time that was postulated to be one of the main contributory factors in senescence [39]. In order to examine this suggestion, the cells were assessed for cellular peroxide production, mitochondrial mass and mitochondrial membrane potential (MMP) by DHR123, NAO and Rho123 staining, respectively. Nonfluorescent dye DHR123 selectively accumulates in mitochondria, where it can be oxidized by mitochondria-derived ROS to a fluorescent rhodamine derivative. As seen in Fig. 5 C, at 24 h post H_2_O_2_ treatment the cellular peroxides levels were almost 2-fold higher than in the control cells and then gradually enhanced for 7 days, indicating that, in the senescent cells, there are permanently elevated ROS levels derived from mitochondria. Next, to determine whether H_2_O_2_ may cause the proliferation of mitochondria, NAO dye to monitor the mitochondrial mass was used. The relative NAO intensity of H_2_O_2_-treated cells gradually increased in time and, on 7 days post-treatment, it was found to be 2.5-fold higher than that of the control cells (Fig. 6 A, B). These results indicate that H_2_O_2_ can promote an increase in the number of mitochondria in hMESCs in a time-dependent manner. To analyze, whether the extra mitochondria in H_2_O_2_-treated cells were functional at the same time, MMP of cells was measured. In treated cells, the relative intensity of Rho123 fluorescence was substantially higher than that of the control cells (Fig. 6 C, D). Importantly, the increase in MMP correlated with corresponding increase in the mitochondrial mass over the entire observation period. In addition, both characteristics also correlated well with elevated cellular peroxide production.

Taken together, these results clearly indicate that oxidative stress induced by the sublethal H_2_O_2_ led to an increase in the amount of functional mitochondria. We assume that an increased mitochondrial activity may be responsible, at least in part, for long-term ROS production observed in the senescent hMESCs.

### The role of p38 in the regulation of H_2_O_2_-induced premature senescence of hMESCs

**Figure 7:**
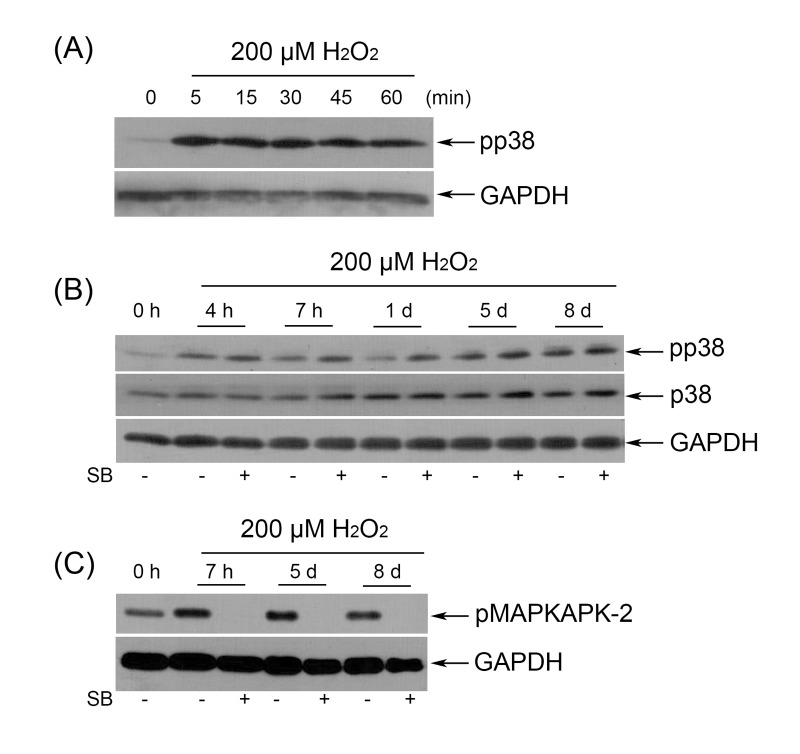
(**A**) Immunoblot analysis of p38 phosphorylation during 1 h H_2_O_2_ treatment. (**B**) SB had no effect on long-term p38 phosphorylation as detected by Western blot. (**C**) Selective inhibition of p38 activity abolished phosphorylation of MAPKAPK-2 during the whole observation period. Representative results of three independent experiments are shown. GAPDH was used as loading control.

First, we examined whether p38 is activated by sublethal doses of H_2_O_2_ in hMESCs. As shown in Fig. 7 A, 200 µM H_2_O_2_ induced a significant increase in p38 phosphorylation within 1 h of treatment, whereas in untreated cells p38 phosphorylation was undetectable. Interestingly, H_2_O_2_ maintained the elevated p38 phosphorylation up to 8 days without affecting steady-state protein levels of p38 (Fig. 7 B). Phosphorylation of MK-2, a natural substrate of p38, was detected at the same time (Fig. 7 C).

To explore the role of p38 in regulation of premature hMESCs senescence, we employed SB203580 (hereafter SB), a specific inhibitor of the p38 MAPK pathway. SB is a small molecule that displaces ATP from the ATP-binding pocket of p38, thereby preventing from phosphorylation of p38 targets, in particular MK-2, without preventing p38 phosphorylation itself [40]. As expected, in H_2_O_2_-treated cells, p38 phosphorylation was unaffected by SB (Fig. 7 B), nonetheless, MK-2 phosphorylation was abolished throughout the 8-days period of the experiment (Fig. 7 C).

**Figure 8:**
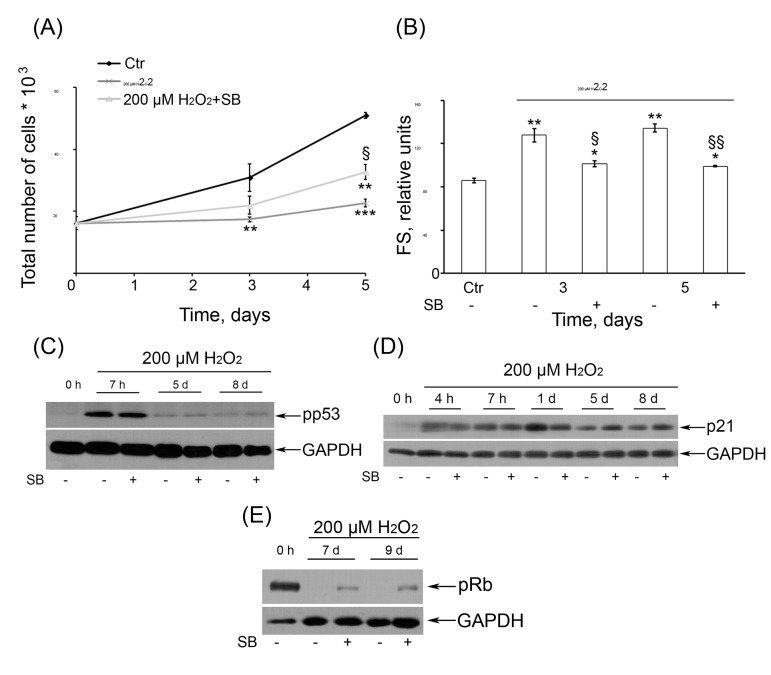
(**A**) SB rescued the cell proliferation of H_2_O_2_-treated hMESCs. Cell number was determined daily by FACS (M ± SD, *N* = 3, **p<0.005, ***p<0.001, versus control, §p<0.05, versus H_2_O_2_-treated cells). (**B**) SB partially prevented H_2_O_2_-induced increase of cell size. Forward scatter (FS) reflects the average cell size. M ± SD, *N* = 3, *p<0.05, **p<0.005, versus control, §p<0.05, §§<0.005, versus H2O2-treated cells. (**C**) Inhibition of p38 activity had no effect on long-term activation of pp53 or (**D**) p21. (**E**) An impact of inhibition of p38 kinase activity on pRb phosphorylation status.

Selective inhibition of p38 kinase activity with SB prevented the increase of the size of H_2_O_2_-treated cells (Fig. 8 B) but just slightly reduced their SA-β-Gal activity (data not shown), indicating some modulation of the senescence phenotype of H_2_O_2_-treated cells. Because the growth arrest was shown to be the major mechanism of the integral growth-inhibitory effect of H_2_O_2_ in hMESCs under our experimental conditions [36], we next checked the role of p38 in the regulation of cell proliferation. As presented in Fig. 8 A, blocking of p38 with SB led to marked increasing the number of proliferating cells compared with H_2_O_2_-stimulated cells. In order to relieve the proliferation block of the senescent cells to a great extent, in the separate experiments, SB was added at 24 or 48 h post H_2_O_2_ treatment unlike the routine immediate adding after H_2_O_2_ removal. Interestingly, in this case SB had no effect on the proliferative status of senescent cells, albeit was still able to reduce ROS levels (data not shown). Consequently, the p38/MK-2 inactivation could in part prevent the loss of the proliferative potential of pre-senescent hMESCs. In this case the senescence program has already been initiated however the proliferative arrest could yet be reversed. By contrast, suppression of p38/MK-2 activity in the senescent arrested cells was insufficient to resume the proliferation. These findings demonstrate that p38, acting as a negative regulator proliferation of hMESCs in response to oxidative stress, is required for establishing premature senescence, whereas its inhibition may at least in part rescue the cells from senescence induction.

Remarkably, in H_2_O_2_-treated hMESCs, p38 inhibition did not affect phosphorylation status of p53 (Fig. 8 C) and did not prevent p21 protein induction over the entire observation period (Fig. 8 D). These results suggest that p38/MK-2 and p53/p21 signaling pathways can act independently during establishing and maintaining of the premature senescence of hMESCs. On the other hand, suppression of p38/MK-2 in H_2_O_2_-treated cells noticeably elevated the pRb phosphorylation levels, indicating an inactivation pRb (Fig. 8 E). These findings correlate well with an increase in the proliferative potential of cells after common treatment with H_2_O_2_ and SB as compared with H_2_O_2_-treated cells (Fig. 8 A).

To confirm that the effect of SB was specific to p38, we used another p38 inhibitor with an unrelated chemical structure, BIRB796 [41]. According to preliminary results, the observed effect of BIRB796 at concentration of 5µM was similar to the effect of SB, partially preventing both H_2_O_2_-induced growth inhibition and the increase in the size of H_2_O_2_-treated cells, thereby demonstrating that p38 participates in the establishing premature senescence in hMESCs induced by H_2_O_2_.

### p38 MAPK implication in feedback loop via ROS

**Figure 9:**
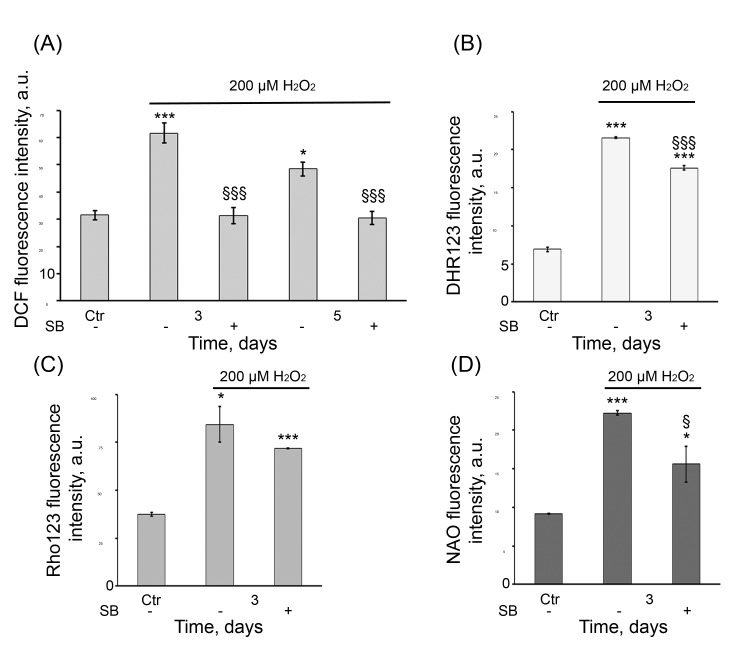
SB diminished ROS production and mitochondrial function in senescent hMESCs (**A**) DCF, (**B**) DHR123, (**C**) Rho123, and (**D**) NAO fluorescence in control (Ctr), H_2_O_2_-treated and (SB+H_2_O_2_)-treated hMESCs at indicated time points as measured by flow cytometry (M ± SD, *N* = 3, *p<0.05, ***p<0.001, versus control, §p<0.05, §§§<0.001, versus H_2_O_2_-treated cells).

As described above, the senescent hMESCs are characterized by persistently elevated ROS levels. We presumed that long-term activation of p38 might be involved in regulation of ROS production. To test this idea, in the SB-treated and untreated senescent cells, the intracellular ROS production measured by H_2_DCFDA staining was evaluated. SB treatment of H_2_O_2_-stimulated cells led to a dramatic drop in the intracellular ROS levels in any of time points tested (Fig. 9 A). Taking into account the fact that increased mitochondria could mediate the elevation of ROS levels in the senescent hMESCs, we next examined whether p38 is able to affect the mitochondrial function. SB treatment of the senescent cells equally reduced the cellular peroxide levels and MMP compared with H_2_O_2_-treated cells as evaluated by DHR123 and Rho123 staining, respectively (Fig. 9 B, C). In addition, in SB-treated cells we observed the similar decrease of mitochondrial mass measured by NAO stain-ing (Fig. 9 D). Overall, the results obtained indicate p38 implication in continued ROS production mediated by in-creased mitochondrial function in the senescent hMESCs.

## DISCUSSION

In the current study, we have examined the molecular mechanism of premature senescence of hMESCs in response to oxidative stress. According to the modern concepts, the process of stress-induced senescence comprises two sequential stages – establishing and maintaining (stabilization) that can be regulated by different mechanisms, depending upon the specific stimuli used, the cell context and other factors. Understanding of the interplay between various signaling pathways that provide the control either stage would have significant therapeutic implications and should be highly useful to determinate the strategy for the reversal of cellular senescence.

DDR activation induced by ionizing radiation, chemotherapeutic drugs or oxidative stress has been investigated in different stem cell types, including bone marrow-derived hMSCs [8], hematopoietic stem cells (HSC) [14] and embryonic stem cells [42]. In the literature, the similar studies performed on hMESCs have not been described thus far. The results presented herein demonstrate that, in hMESCs subjected to sublethal oxidative stress, H_2_O_2_ generated a persistent DDR signaling associated with DNA double-strand breaks (DSBs) which are a signal for activation of ATM and downstream pathways, leading to cell cycle block, as well as the accumulation of DNA foci marked by γH2A.X and phospho-53BP1. Activation of DDR signaling can be a trigger for switching on senescence and it is essential for establishing and maintaining senescent phenotype of cells [15].

We have reported earlier [36] that, in hMESCs, the exogenous H_2_O_2_ caused an irreversible arrest of cell cycle with predominant accumulation of cells in G0/G1-phase. To find out the molecular mechanism, triggering the cell cycle arrest in H_2_O_2_-treated hMESCs, first of all we focused on p53-mediated signaling pathway which may lead to the cell cycle block. It is well documented that the p53/p21 pathway is critical for establishing the replicative senescence of human cells [12, 13], as well as the premature senescence of hMSCs [8] and HSC [14]. However, little is known about which signaling pathways are responsible for the induction of the premature senescence of hMESCs under oxidative stress. In agreement with a canonical p53/p21 model, our results demonstrate that DDR-activated p53 upregulated the CDK inhibitor p21 that, in turn, prevented the phosphorylation and inactivation of pRb. Besides, pRb activity may be controlled by another CDK inhibitor, p16 (INK4a). Previous studies suggested that p16 was crucial for long-term maintaining of senescence of both human fibroblasts [1, 13, 43] and HSC [14]. Interestingly, in these cases p16 was expressed much later than p21, forming a second barrier to prevent the cells from cell cycle re-entering. In contrast, our preliminary data indicate that up-regulation of p16 occurs solely at initial stage of senescence (within 1 h) but not at delayed time period. These findings support the possibility that p16/pRb pathway, in addition to p53/p21, is responsible for establishing the growth arrest, preventing entering the cells into S-phase.

In consideration of strongly decreased p53 activity observed in senescent cells from 3 days, we can speculate that p53 is critical for rather establishing the senescence growth arrest than prolonged maintaining the senescent state of hMESCs. Unlike p53, an inducible p21 expression was persistently upregulated throughout experiment (up to day 21) and was accompanied with the cell cycle arrest [36]. Accordingly, the elevated p21 induction was indispensable to promote the senescence, as well as to maintain this state in hMESCs in response to H_2_O_2_. Previously, it was reported that the activated checkpoint kinase 2 (Chk2) can induce p21 transcription in the absence of functional p53 and that this contributes to Chk2-mediated senescence [44]. It was attractive to suppose that, in our experimental conditions, Chk2 permanently activated by ATM also is able to mediate a long-term p21 induction during senescence. Taken together, our findings definitely demonstrate that, in H_2_O_2_-treated hMESCs, the senescence program is triggered by DDR signaling, activation of which leads to an irreversible cell cycle arrest through p53/p21/pRb pathway.

Further, we tested the role of stress-activated kinase p38 (in complex with MK-2, a direct downstream target of p38) as the most prominent mediator of stress-induced cellular senescence. MK-2 is known to be a negative regulator of cell cycle progression because it is directly responsible for phosphorylation-dependent inactivation of members of the Cdc25 family of phosphatases, which are positive regulators of Cyclin/CDK complexes. Finally, pRb was found to promote stress-induced growth arrest as a downstream molecule of p38 [45]. At present, MK-2 is recognized as a new member of the DNA damage checkpoint kinase family that functions in parallel with Chk1 and Chk2 to integrate DNA damage responses and cell cycle arrest [46]. Previous studies reported that the p38 pathway is implicated in H_2_O_2_-induced senescence of human fibroblasts however the data presented by various groups were controversial. Several authors observed in H_2_O_2_-treated cells the continuous p38 activation [45] while the others demonstrated that transiently elevated p38 kinase activity was reversible down-regulated after H_2_O_2_ removal [47]. Moreover, no activation of p38 was detected in H_2_O_2_-treated fibroblasts, regardless of endogenous p38 expression [48]. By contrast, our results indicate that elevated p38/MK-2 activation in response to H_2_O_2_ was persisted for a long time that suggests the importance of p38/MK-2 pathway in the control of both induction and a long-term maintaining of hMESCs senescence. The fact that the permanent p38/MK-2 activation was accompanied by pRb inactivation argues in favour of the p38/MK-2/pRb pathway that is likely to be mediated by Cdc25 family members. Interestingly, during treatment of hMESCs with H_2_O_2_, p38 activation was accompanied with increasing in phosphorylation of ASK1 at Thr845 (results not presented) that is correlated with ASK1 activity. Therefore, at this stage, we cannot exclude the possibility that ASK1 is involved in up-regulation of p38.

It is noteworthy that inactivation of p38/MK-2 induced by SB did not affect the functional status of p53 and p21, suggesting that p38/MK-2 and p53/p21 pathways are uncoupled however can cooperate to induce an irreversible proliferative arrest, and further to maintain the premature senescence of hMESCs. Generally, this suggestion is supported by a recent report demonstrating that p38 participates in oxidative stress-induced senescence via an alternative ATM-independent pathway, implicating lamin B1 accumulation [49].

The pharmacological inhibition of p38 activation may be considered as a possible strategy for senescence prevention [45, 49]. According to our results, selective inhibition of p38 kinase activity with SB abrogated H_2_O_2_-induced cell enlargement and flattened morphology, but did not produce any significant effect on SA-β-Gal activity. At the same time, SB treatment of hMESCs allowed to avoid an irreversible cell cycle arrest in response to H_2_O_2_ however the recovery of proliferation was incomplete. This may point at the probable dissociation of hallmarks of senescence - senescent morphology, RP and SA-β-Gal staining. Likewise, elimination of cyclin D1 (a universal marker of cellular senescence) by specific inhibitors of the MEK/ERK pathway did not affect at least three classical hallmarks of senescence: loss of RP, senescent morphology and SA-β-Gal staining [20]. The findings that inhibition of p38 partially suppressed the H_2_O_2_-induced senescent phenotype of hMESCs, as well as prevented the proliferation arrest indicate that activation of p38 contributes to H_2_O_2_-induced cellular senescence. A plausible explanation for the partial effect of SB on growth arrest is that SB being a specific inhibitor of p38α and p38β cannot suppress the redundant γ and δ isoforms of p38 [50]. In addition, SB was reported to produce antiproliferative effect related to inhibition of pRb phosphorylation [51]. Thus, suppression of p38 kinase activity can at least in part rescue stressed hMESCs from the cell cycle arrest and entering premature senescence induced by H_2_O_2_. It is of noted, that the permanent growth arrest correlated with ROS accumulation during development of senescence.

Our attempts to find out the possible reasons for maintaining the H_2_O_2_-induced senescence of hMESCs led to the observation that there is the interplay between permanently elevated ROS and the persistence of DDR signaling. In fact, the senescent cells displayed the persistent accumulation of DNA damage foci marked by p53BP1 and γH2A.X associated with pATM, as well as continuously increased levels of both intracellular ROS and mitochondrial peroxides. These findings are in line with previous study, presuming that the feedback loop between DDR and ROS production is necessary and sufficient to maintain senescent growth arrest during establishment of irreversible senescence [22]. Consistent with supposed mechanism, the senescent cells persistently accumulate senescence-associated DNA damage foci (SDFs), which contain proteins associated with DNA damage, particularly γH2A.X and p53BP1. The prolonged DDR activation results in up-regulation of p53 and p21 that may induce the increase in intracellular ROS levels. All together, the intracellular ROS are able directly to damage DNA and thus sustain DDR in an active state.

To clarify the mechanism which may regulate the feedback loop, we utilized an inhibitor analysis of long-term ROS production. In senescent hMESCs, the specific inhibition of p38/MK-2 activity by SB had a pronounced negative effect on the intracellular (cytosolic) ROS production while the mitochondrial ROS production was diminished just in part. Similarly, mitochondrial mass and mitochondrial membrane potential were partially decreased. It is important to note that in senescent hMESCs preserving the metabolic activity we revealed the significant increase in the amount of functional mitochondria which might be responsible, at least in part, for long-term ROS production. These findings collectively allow speculation of p38/MK-2 involvement in modulation of intracellular ROS that are critical for maintaining feedback loop during senescence of hMESCs. Although our results suggest that the activated p38/MK-2 complex may regulate ROS generation via functional mitochondria, it is most likely that such regulation is indirect. Accumulating evidence pointed to an important role for TGFβ/TGFβ receptor and GADD45 in mitochondrial ROS production mediated by p38 [22, 28].

**Figure 10:**
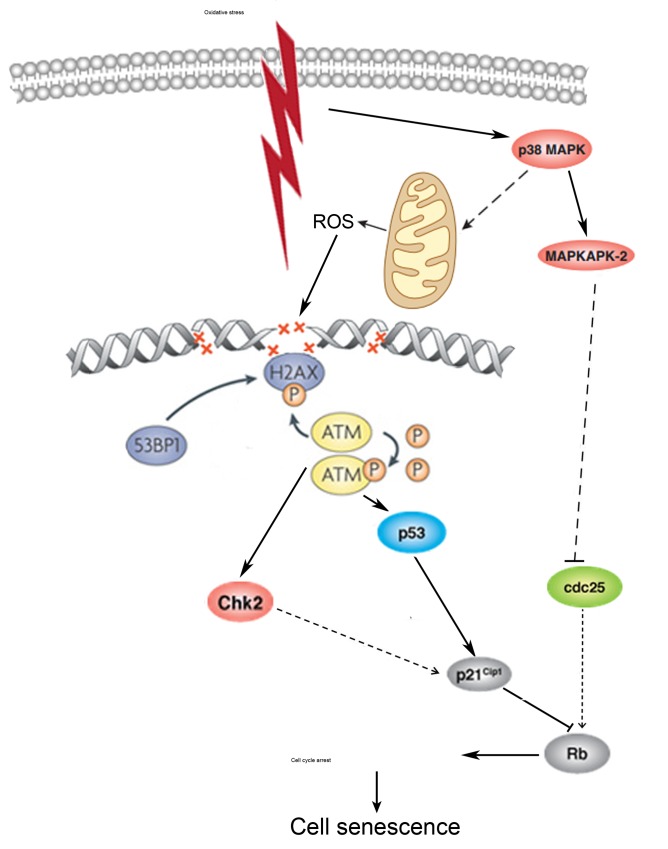
A pathway interaction scheme displaying the proposed molecular mechanism of premature sense-cence of hMESCs under oxidative stress

In conclusion, the present study is the first to elucidate the molecular mechanism of premature senescence of hMESCs under oxidative stress. The induction of senescence includes a prompt activation of response to DNA damage induced by H_2_O_2_ and following signal transduction through p53/p21 and p38/MK-2 pathways which are necessary and sufficient to establish the irreversible cell cycle arrest that is typical of senescence. We believe the prolonged induction of p21 as well as elevated activation of p38/MK-2 also might be indispensable to maintain persistent proliferative block in senescent cells. Additionally, p38 which may regulate both intracellular and mitochondrial ROS production is possibly involved in senescence stabilization via the feedback loop that provides sustained activation of DDR signaling (Fig. 10).

The main properties of hMESCs - the capacity for self-renewal, multilineage differentiation and noninvasive isolation procedures bring them to the cutting edge of regenerative medicine. Although hMESCs trans-plantation for treatment of heart failure, myocardial infarction, Duchenne Muscular Dystrophy has been successfully applied, it should be taken into account the possibility of premature senescence of these adult stem cells in stress conditions with following loss of the regenerative potential and, consequently, of the capability to regenerate the injured tissues. Understanding the mechanism of premature senescence of hMESCs induced by H_2_O_2_ should provide more effective strategies in transplantation of these cells into the recipients with age-related disorders inherently associated with increased levels of oxidative stress.

## METHODS

### Cell culture

Human mesenchymal stem cells isolated from desquamated endometrium in menstrual blood (hMESCs, line 2304) as described previously [52] were cultured in complete medium (DMEM/F12 (Gibco BRL, USA) supplemented with 10% FBS (HyClone, USA), 1% glutamax (Gibco BRL, USA), 1% gentamycin) at 37°C in humidified incubator, containing 5% CO_2_. hMESCs had a positive expression of CD73, CD90, CD105, CD13, CD29, and CD44 markers; expression of the hematopoietic cell surface antigens CD19, CD34, CD45, CD117, CD130, and HLA-DR (class II) was absent. Besides, hMESCs partially (over 50%) expressed the pluripotency marker SSEA-4 but not Oct-4. Multipotency of isolated hMESCs was confirmed by their ability to differentiate into other mesodermal cell types, such as osteocytes and adipocytes. Immunofluorescent analysis revealed the expression of the neural precursor markers nestin and beta-III-tubulin that suggests a neural predisposition of hMESCs. Cells were characterized by high rate of cell proliferation (doubling time 22-23 h) and high cloning efficiency (about 60%). To avoid complications of replicative senescence, cells at early passages (between 5 and 9 passages) were used in all experiments.

### Cell treatments

Cells were harvested by trypsinization and plated at a density of 15*10^3^ cells per cm^2^. For microscopy experiments, cells were grown on glass coverslips. H_2_O_2_ treatment was performed on sub-confluent cells to avoid variability of H_2_O_2_ toxicity. H_2_O_2_ stock solution in serum-free medium was prepared from 30% H_2_O_2_ (Sigma, USA) just before adding. Cells were treated with 200 µM H_2_O_2_ for 1 h, then washed twice with serum-free medium to remove H_2_O_2_, and either re-cultured in fresh complete medium for various durations as specified in individual experiments or, when indicated, treated with 5 µM SB203580 (Sigma, USA) for 40 min at 37°C. As rule, SB was added in the culture medium immediately after H_2_O_2_ treatment, and then it has been added daily to avoid the degradation of inhibitor.

### Measurments of ROS, cellular peroxides, mitochondrial membrane potential and mitochondrial mass by FACS and confocal microscopy

Adherent cells treated with H_2_O_2_ were rinsed twice with PBS and probed with fluorescent dyes (all from Molecular Probes) prepared in serum-free medium. To detect intracellular ROS, cells were stained with 10 µM 2',7'-dichlorodihydrofluorescein diacetate (H_2_DCFDA) for 20 min at 37°C. Cellular peroxide levels were assessed by staining with 30 µM dihydrorhodamine 123 (DHR 123) for 30 min at 37°C. Mitochondrial membrane potential was measured after cell staining with 10 µM Rhodamine 123 (Rho123) for 30 min at 37°C. Mitochondrial mass was determined after cell staining with 10 µM 10-n-Nonyl-Acridine Orange (NAO) for 10 min at 37°C. After incubation, cells were analyzed either by FACS or confocal microscopy. For FACS cells were harvested by trypsinization and immediately analyzed on a Coulter EPICS XL Flow Cytometer (Backman Coulter, USA). All parameters were measured as FL1 fluorescence. For confocal scanning imaging, cells grown on coverslips were moved to the imaging system of scanning confocal microscope Leica TCS SP5 MP (Leica Microsystems Inc., Bannockburn, IL). Single focal plane images were merged and analyzed with standard Leica LAS AF Software (Leica Microsystems). Fluorescent cells were viewed with 20x objective (HCX APO CS20x/0.70; Leica Microsystems). For all dyes fluorescence was excited with 488 nm laser and observed in green spectral region (510-560 nm). Sampling frequency was set to 0.033 fps for fluorescent imaging. Fluorescent intensity was recorded in the regions of interest from 20-35 cells. After baseline collection (5 min), cells were treated with 200 µM H_2_O_2_ for 55 min. Next measurements were done at DIV5 (after cell incubation with H_2_O_2_ during 60 min at DIV1).

### Immunofluorescence

H_2_O_2_-treated cells grown on coverslips were fixed with 4% formaldehyde (15 min), permeabilized with 0.1% Triton X-100 (10 min) and blocked with 1% BSA (1 h). Cells were incubated with primary antibodies – a rabbit polyclonal antibodies against γH2A.X (Ser139) (Abcam), phospho-53BP1 (Ser1778) (Cell Signaling), phospho-p53 (Ser15) (Cell Signaling) and mouse polyclonal antibodies against phospho-ATM (Ser1981) (Thermo Scientific) overnight at 4°C and secondary antibodies – Alexa Fluor 568 goat anti-rabbit or Alexa Fluor 488 goat anti-mouse (Invitrogen) for 1 h at room temperature after extensive washing with PBS/0.1% Tween 20 between each step. The slides were counterstained with 1 µg/ml DAPI (Sigma) and mounted using 2% propyl gallate. A Zeiss Axiovert 200M fluorescence microscope (Carl Zeiss) equipped with a digital camera DFC 420C (Leica) utilizing Adobe Photoshop software was used to view and acquire images.

### Western blotting

Western analysis was performed as described previously [53]. Primary antibodies (all from Cell Signaling) against the following proteins were used: p21Waf1/Cip1, phospho-ATM (Ser1981), p38αMAPK, phospho-MAPKAPK-2 (Thr334), glyceraldehyde-3-phosphate dehydrogenase (GAPDH, clone 14C10), phospho-p53 (Ser15), phospho-Rb (Ser807/811), phospho-p38MAPK (Thr180/Tyr182).

### RT-PCR assay

To analyze gene expression, total cellular RNA was isolated with RNesy Micro Kit (Qiagen) according to manufacturer's instructions. cDNA synthesis was performed with 1 μg of total RNA using RevertAid H Minus First Strand cDNA Synthesis Kit (Fermentas) according to manufacturer's instructions. Specific genes were amplified by Taq DNA polymerase (Fermentas) with C1000 TouchThermal Cycler amplifier (Bio-Rad Laboratories). The program was described earlier [30]. Primers *p21Waf1/Cip1* and *beta-actin* were obtained from SYNTOL (Russia). The electrophoresis of amplified products was performed in 2% agarose gel with TAE buffer and ethidiumbromide. 100 kb DNA ladder (Fermentas) was used as molecular weight marker. Amplified products were visualized in UV-light (302 nm) with transilluminator and registered with a digital Canon camera.

### FACS analysis of cell viability and cell size

Adherent cells were rinsed twice with PBS and harvested by trypsinization. Detached cells were pelleted by centrifugation. Finally, detached and adherent cells were pooled and resuspended in PBS. 50 µg/ml propidium iodide (PI) was added to each sample just before analysis and mixed gently. Samples were analyzed on a Coulter EPICS XL Flow Cytometer (Backman Coulter). The cell size was evaluated by cytometric light scattering of PI-stained cells with using Win MDI program version 2.8. To discriminate the live and dead cells, two-parameter histogram was used (FL4LOG vs. FSLOG). Analysis of each sample (at least 10,000 cells) was performed for 100 sec with high sample delivery.

### Statistics

All data are presented as the mean and standard deviation of the mean from at least three separate experiments performed. Statistical differences were calculated using the Student's t-test and considered significant at *,§ p< 0.05; **,§§ p< 0.005; ***,§§§ p<0.001.
